# Comparative Evaluation of Heavy Metals in Patients with Rheumatoid Arthritis and Healthy Control in Pakistani Population

**Published:** 2017-05

**Authors:** Shazia IRFAN, Asima RANI, Naila RIAZ, Muhammad ARSHAD, Syed KASHIF NAWAZ

**Affiliations:** 1. Dept. of Zoology, University of Sargodha, Sargodha, Pakistan; 2. Principal, University of Education, Lower Mall Campus, Lahore, Pakistan

**Keywords:** Rheumatoid arthritis, Heavy metals, Serum, Pakistan

## Abstract

**Background:**

Exposure to heavy metals in development of many diseases has been investigated previously, specially created by oxidative stress. The etiology of Rheumatoid arthritis (RA) is still not fully understood but oxidative stress created by heavy metals may have role in development of RA. The aim of present study was to compare serum level of heavy metals in RA and healthy control individuals.

**Methods:**

Blood samples of 100 RA patients were collected from different hospitals in district Sargodha, Punjab, Pakistan and 100 control individuals from Dec 2013 to May 2014.The serum samples were analyzed for determination of Pb, Cd, Cr and Ni through Atomic absorption spectrophotometer (AA 6600 Shimadzu).

**Results:**

Statistically highly significant difference was observed between RA patients and healthy control individuals for Pb, Cd, Cr, and Ni level (*P*<0.01). The difference between the means of both sexes was not significant for Pb and Cd concentrations (*P*>0.01). For Cr the difference between the means of both sexes was statistically not significant in RA +ve patients and highly significant difference was observed between both sexes in healthy control group (*P*<0.01). The difference between the means of both sexes for Ni was statistically non-significant in healthy control group while significant difference was observed between both sexes in RA +ve group (*P*<0.05). Statistically non-significant difference for Pb, Cd, Cr and Ni level was found among the all three age groups of RA and healthy control individuals (*P*>0.01).

**Conclusion:**

Concentration of heavy metals in serum samples of RA patients and healthy control individuals differ significantly, which shows that heavy metals may contributes towards development of RA.

## Introduction

One of inflammatory form of arthritis is Rheumatoid arthritis (RA) in which a synovial membrane is attacked and cause swelling, pain and stiffness of joint. It is typically a progressive disease leading to joint destruction and functional disability. The exact cause of RA is still unknown. There are many contributing factors of RA like SNPs in different genes, drugs, chemicals, bacteria, some viruses like hepatitis C virus, Epstein bar virus and metals (1, 2). Among the many contributing agents proposed to take part in the pathogenesis of this condition heavy metals also are investigated previously.

Our environment is so much polluted that every day particles of different heavy metals easily pass in to our system. Exposure to heavy metals plays a role in the induction or exacerbation of several autoimmune diseases (3). Heavy metals influence the development of autoimmunity and one of most important autoimmune disorder is RA. Mercury (Hg), cadmium (Cd), arsenic (As), lead (Pb), antimony (Sb), tin (Sn), cobalt (Co), manganese (Mn) and chromium (Cr) exposure has been considered important in the development of RA (4). Although the mechanism of metal ion toxicity is not fully known but it is evident that they can generate reactive oxygen species (ROS), such as superoxide ions (O2-), hydrogen peroxide (H_2_O_2_), hydroxyl radical (OH), and nitrogen oxide (NO) through Fenton/ Haber-Weiss chemistry (5). The production of ROS play role in many human diseases including degenerative lung and heart conditions, Alzheimer disease, RA and aging (6).

Pb is a most common heavy metal found throughout the environment and it poses a significant health risk if too much enters the body. Many studies showed that Pb is main culprit of oxidative stress as it is shown to deplete antioxidant proteins and induce the production of ROS and RNS (7, 8).

Cr has significant importance in altering the immune response causing immunostimulatory or immunosuppressive processes. The reduction of Cr (VI) to Cr (III) results in the formation of ROS that create oxidative stress and cause oxidative tissue damage (9) and this oxidative stress is major contributor towards development of RA.

One of toxic heavy metal is Cd that has no biological function. It interferes with calcium metabolism and cause replacement of calcium in the bones and contribute towards development of osteoporosis, osteomalacia, stones in ureter and kidney, hypercalcuria and RA. It also accumulates in the joints and play role in development of osteoarthritis (10). Some other mechanisms of Cd-mediated pro-oxidative activity include: 1) inhibition of superoxide dismutase (11); 2) bonding to sulfhydryl groups, depleting glutathione and protein sulfhydryl, thereby compromising intracellular anti-oxidative defenses (12).

Ni is also a metal and it cause depletion of glutathione enzyme and protein-bound sulfhydryl group and cause production of ROS such as O2-, H_2_O_2_ and OH. Ni toxicity also affects enzymatic and non-enzymatic antioxidants functioning (13). Uncertainty exists regarding level of heavy metals like Pb, Cd, Cr and Ni in pathogenesis of RA as some authors reported high and some reported low level of these metals. Therefore, it is needed to reexamine the status of the selected metals in RA patients. A little information is present regarding gender base and age wise distribution of these metals in RA patients. Therefore, the objective of present study was to evaluate comparative distribution and correlation of heavy metals in RA patients and control.

## Materials and Methods

### Sample collection

All procedures were in agreement with the declaration of Helsinki. The Advance Research and Study Board, University of Sargodha has approved the protocol of present study. Ethical Committee, University of Sargodha granted permission for the start of research work.

The blood samples of 400 individuals including RA patients and control were collected after taking proper consent and completion of ethical criteria. Blood samples of 100 RA patients were collected from different hospitals in district Sargodha, Punjab, Pakistan and 100 control individuals from Dec 2013 to May 2014. The questioner was filled regarding data related to patients like age and gender was collected from laboratories of hospitals.

### Treatment of Samples

Five ml of blood was collected from each sample using BD syringes. Blood samples were centrifuged at 10000 rpm for 3 min to separate serum. Serum was collected with the help of micropipette and put into eppendorfs. After that, these tubes were marked and stored below 4 ^°^C before further processing. With the help of micropipette, 1 ml serum was taken and transferred to flask.

### Wet Acid digestion

Nitric acid+ hydrogen peroxide (HNO3 + H_2_O_2_) was added in the ratio 4:1 (4ml HNO3 + 1ml H_2_O_2_) and left overnight for incubation. On hot plate, samples were heated until they were near to dry. These samples were then removed from the hot plate and 2 ml of H_2_O_2_ was added to them. This process was repeated many times until the sample becomes water clear. The contents of the flask were filtered and collected in 50 ml volume-tric flask in order to make the volume with deionized water. Solutions were transferred into marked Teflon bottles (14).

After wet acid digestion, the blood samples were analyzed for determination of Pb, Cd, Cr and Ni through Atomic absorption spectrophotometer (AA 6600 Shimadzu). Standards were used for the standard curve formation and estimation of metals in the samples.

For method validation, linearity, limit of detection (LD) and limit of quantification (LQ), accuracy and intra and inter precision of all assay were determined. Linearity was evaluated through graphical representation of concentration versus absorbance. In case of Pb and Cr three calibration curves with five different concentrations of standard solutions (3, 6, 9, 12 and 15 ug/L), for Cd (0.2, 0.4, 0.6, 0.8 and 0.10ug/L) and Ni (35, 60, 95,120 and 150ug/L) were prepared. Linearity was evaluated through the calculation of the Pearson (R2) coefficient of correlation through the analysis of residues with the coefficient of determination (R). The results obtained for all four metals were close to 1.00. The linearity is acceptable, since a value of R2 or R > 0.995 is regarded as acceptable (IUPAC, 1999).

The assay sensitivity was also checked. The solutions prepared for estimating the linearity, were used to evaluate the LD and LQ. LD was calculated as three deviation standard and LQ was calculated as ten deviation standard from six independent replicates of sample corresponding to the first point of the calibration curve. The LD for Pb, Cd, Cr and Ni was 0.04ug/L, 0.2ug/L, 0.26ug/L and 21.5ug/L and the LQ was 0.12ug/L for Pb, 0.6ug/L for Cd, 0.78ug/L for Cr and 64.5ug/L for Ni. To evaluate the accuracy, individual standards of work were prepared to concentrations of 9ug/L for Pb and Cr. For Cd and Ni the used concentration was of 0.4ug/L and 95ug/L, which represent the average value of the concentrations used for the calibration curves. The results obtained of the individual standards of Pb, Cd, Cr and Ni indicate that the accuracy obtained is acceptable as the recovery rates for the four metals fluctuated in a range of 97.87% to 101.01% with an overall average of 99.28% recovery.

Repeatability (intra-day) precision was calculated by making five determinations of standard solution with in same day and intermediate precision (inter-day) were also assessed by analyzing working standard solutions on five different days. The intra-day CV was below 5% and inter-day CV was below 10% for all studied elements.

### Statistical Analysis

Data was processed using 13 (Chicago, IL, USA). One way ANOVA and two sample T test was performed to depict statistical differences. The results were presented as Mean ± Standard deviation (SD) and a *P*-value of <0.01 was considered as significant. Correlation studies were carried out by using Pearson’s correlation coefficient.

## Results

This study was designed to compare concentration of heavy metals between healthy control individuals and RA. Atomic absorption spectrophotometry was used for the analysis.

**Fig. 1:**
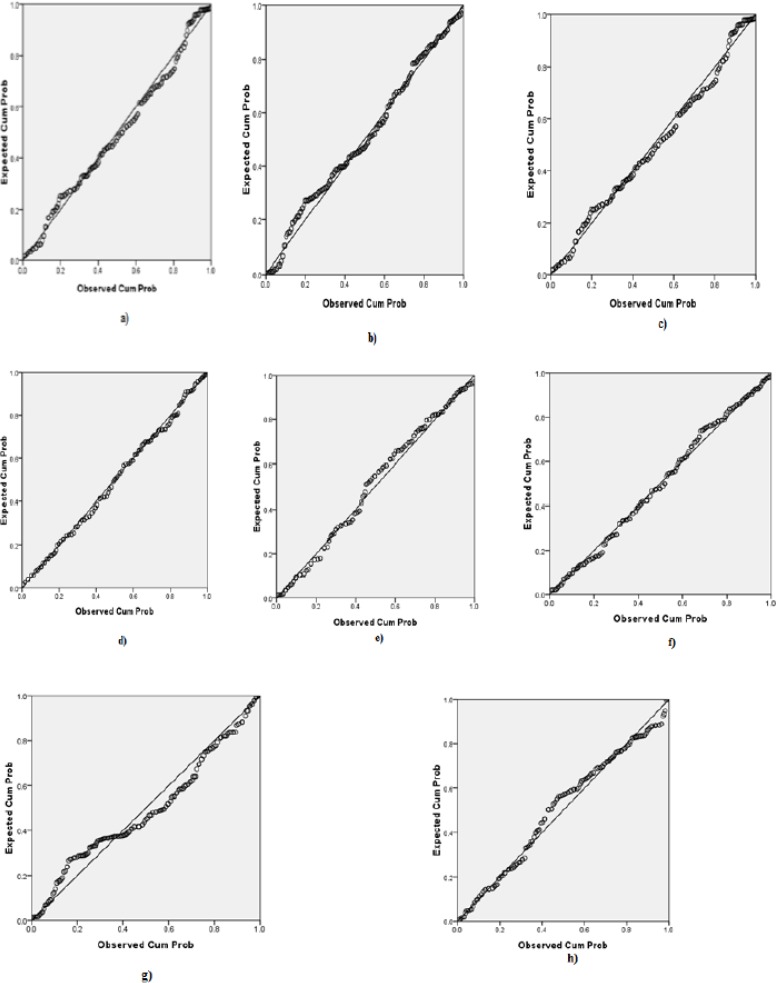
Normal probability plot of heavy metals. a) Pb (control), b) Pb (RA), c) Cd (control), d) Cd (RA), e) Cr (control), f) Cr (RA), g) Ni (control), h) Ni (RA)

The normality of data can be checked through different methods but the most common method used for checking the normality of the data is Normal probability plot (NPP). The NPP was applied to data, which showed normal distribution of data, however little bit departure of normality can be ignored (Fig. 1). Parametric test is applied for statistical analysis.

Statistically highly significant difference was observed between RA +ve patients and healthy individuals for Pb, Cd, Cr, and Ni level (*P*<0.01) (Table 1).

**Table 1:** Mean concentration (ug/L) of heavy metals in serum samples of control and RA patients (n=200)

**Metals**	**Studied Group**	**Mean ±SD**	**S.E**	**Comparison of Significance**	
				***t*-test**	***P*-value**
Pb	*Control*	2.1904±1.1433	0.08084	11.477	0.000**
	*RA*	5.7333±4.318	0.30533		
Cd	*Control*	0.2845±0.75959	0.05371	19.278	0.000**
	*RA*	1.767± 0.7981	0.05644		
Cr	*Control*	0.2601±0.1751	0.01238	18.92	0.000**
	*RA*	3.0817±2.119	0.14985		
Ni	*Control*	112.53±11.067	0.78256	−35.619	0.000**
	*RA*	41.19±27.27	1.92		

The mean level of Pb in RA +ve and healthy control individuals was 5.73± 4.31 ug/L and 2.1904±1.14ug/L, respectively while mean concentration of Cd in RA +ve and healthy control individuals was 1.767± 0.7981ug/L and 0.2845±0.75959 ug/L respectively. Mean concentration of Cr was 3.0817±2.119 ug/L in RA +ve patients while in healthy control individuals mean level was found to be 0.2601±0.1751 ug/L, respectively. Similarly, mean concentration of Ni was 41.19±27.27 ug/L in RA +ve patients while in healthy control individuals mean level was found to be 112.53±11.067 ug/L, respectively. Table 2 depict gender wise mean level of heavy metals within RA +ve and healthy control individuals (Fig. 1 c and d) depicts low level of Pb, Cr and Ni was found in male individuals in RA +ve patients as compared to females while Cd level was high in male RA +ve patients. In case of healthy control individuals Cd level was found to be low while Pb, Cr and Ni level was high in male individuals as compared to females.

**Table 2:** Gender wise mean concentration (ug/L) of heavy metals in serum samples of control and RA patients

**Studied group**	**Metals**	**Gender**	**n**	**Mean ±SD**	**S.E**	**Comparison of Significance**	
						***t*-test**	***P*-value**
RA n=200	Pb	Male	89	5.5623± 4.1023	0.43484	−501	0.617 ^NS^
		Female	111	5.8704±4.49724	0.42686		
	Cd	Male	89	1.7781±0.69833	0.07402	0.176	0.861 ^NS^
		Female	111	1.758±0.87296	0.08286		
	Cr	Male	89	2.6466±1.77825	0.18849	−2.639	0.009**
		Female	111	3.4306±2.3062	0.2189		
	Ni	Male	89	36.130±26.302	2.788	−2.377	0.018*
		Female	111	45.2496±27.2799	2.60892		
Control n=200	Pb	Male	134	2.2973±1.295	0.11189	1.898	0.059 ^NS^
		Female	66	1.9731±0.7055	0.08684		
	Cd	Male	134	0.2173±0.525	0.04541	−1.792	0.075 ^NS^
		Female	66	0.4208±1.083	0.13331		
	Cr	Male	134	0.2692±0.1605	0.01387	1.053	0.294 ^NS^
		Female	66	0.2415±0.2014	0.0248		
	Ni	Male	134	113.21±7.4323	0.64205	1.240	0.217 ^NS^
		Female	66	111.15±16.09	0.633		

The difference between the means of both sexes was non-significant for Pb and Cd concentrations (*P*>0.01), while for Ni the difference between the means of both sexes was statistically significant (*P*<0.05) and for Cr concentration highly significant difference was observed between both sexes in RA +ve patients (*P*<0.01). The difference between the means of both sexes for Pb, Cd, Cr and Ni was statistically non-significant in healthy control group (*P*>0.01).

RA+ve and healthy individuals were also categorized into three age groups 20–35, 36–50 and 51–60 yr of age, respectively. Statistically non-significant difference for Pb, Cd and Cr level was found among the all three age groups of RA+ve and healthy control individuals (*P*>0.01) as shown in Table 3. In case of Ni, statistically significant difference was found in healthy control individuals (*P*<0.05) while non-significant difference existed among the all three age groups in RA +ve patients (*P*>0.01).

**Table 3:** Age wise mean concentration (ug/L) of heavy metals in serum samples of control and RA patients

**Metal**	**Age Group**	**20–35**		**36–50**		**51–60**	
**Pb**	**Studied Group**	**Mean ±SD**	**S.E**	**Mean ±SD**	**S.E**	**Mean ±SD**	**S.E**
	*RA*	2.306± 0.90 ^NS^	0.17	2.309 ±1.34 ^NS^	0.129	1.943±0.76 ^NS^	0.094
	*Control*	2.029±1.01 ^NS^	0.15	2.22±1.283 ^NS^	0.11	2.28±0.77 ^NS^	0.12
**Cd**	*RA*	0.198±0.353 ^NS^	0.069	0.35±0.898 ^NS^	0.086	0.203±0.6 ^NS^	0.074
	*Control*	0.29±0.704 ^NS^	0.104	0.207±0.638^NS^	0.05	0.515±1.07^NS^	0.174
**Cr**	*RA*	0.215±0.136 ^NS^	0.02	0.258±0.174 ^NS^	0.016	0.280±0.18^NS^	0.02
	*Control*	0.288±0.20 ^NS^	0.03	0.266±0.16 ^NS^	0.015	0.20±0.153 ^NS^	0.024
**Ni**	*RA*	115.37±7.13 ^NS^	1.39	112.6±8.58 ^NS^	0.82	111.1±15.2^NS^	1.88
	*Control*	108.5±17.903*	2.66	113.2±8.07*	0.74	114.9±6.74*	1.094

N = number of observations (respondents)

NS = Non-significant (P>0.05);

* = Significant (*P*<0.05);

** = highly significant (*P*<0.01)

Correlation coefficient matrix of heavy metals in serum samples of the RA+ve and healthy control individuals were analyzed (Table 4).

**Table 4:** Correlation coefficient matrix of Heavy metals in serum of the control and RA patients

**Studied Group**	**Characters**	**Age**	**Gender**	**Pb**	**Cd**	**Cr**	**Ni**
**RA n=200**	**Gender**	.146*					
		.039					
	**Pb**	.026	.036				
		.716	.617				
	**Cd**	−.003	−.012	.248**			
		.966	.861	.000			
	**Cr**	.016	.184**	−.002	.189**		
		.826	.009	.974	.007		
	**Ni**	.003	.167*	.054	−.161*	.106	
		.961	.018	.451	.023	.135	
**Control n=200**	**Gender**	.005					
		.943					
	**Pb**	.073	−.134				
		.303	.059				
	**Cd**	.087	.126	−.012			
		.219	.075	.868			
	**Cr**	−.148*	−.075	.103	−.063		
		.037	.294	.146	.375		
	**Ni**	.193**	−.088	−.039	−.016	−.214**	
		.006	.217	.582	.827	.002	

Upper values indicated Pearson’s correlation coefficient; Lower values indicated level of significance at 5% probability.

* = Significant (*P*<0.05);

** = Highly significant (*P*<0.01)

In RA+ve patients positive correlation exists between Pb_Age, Pb_gender, Cr_age, Ni_age, Ni_Pb and Ni_Cr while negative correlation exist between Cd_age and Cd_gender. Significantly positive correlation exist between gender_age and Ni_gender while significant but negative correlation exists between Ni_Cd (*P*<0.05). Statistically highly significant but positive correlation exists between Cr_gender, Cr_Cd and Pb_Cd (*P*<0.01). Similarly in healthy control individuals positive correlation exists between gender_age, Pb_age, Cd_age, Cd_gender and Cr_Pb while negative correlation exist between Pb_gender, Cd_Pb, Cr_gender, Cr_Cd, Ni_gender and Ni_Pb. Statistically, highly significant negative correlation exists between Ni_Cr while highly significant but positive correlation exists between Ni and age.

## Discussion

Pb and Cd stimulate the production of cytokines and Pb may interfere with antigen presentation by inhibiting specific Th1 lymphocytes stimulation while promoting presentation to Th2 lymphocytes (15, 16). Chronic exposure to heavy metals, especially Pb, Hg and Cd affects the immune system as a result immune system attacks on its self-molecules, which can lead to RA and other joint diseases (17).

Highly significant difference exists between RA patients and healthy control group for Pb, Cd and Cr concentration when our results were compared with the value found in literature. High level of Pb, Cd and Cr was reported in RA patients (18). Pb has low level in serum of RA patients as compared to healthy control individuals while Cd and Cr were normal in range in both groups (19, 20). Age group comparison and gender base comparison of Pb and Cd in RA patients as well as in healthy control individuals showed non-significant difference (21).

Ni is also essential element as being component of an important antioxidant enzyme superoxide dismutase 3 (22) which catalyze the dismutation of two superoxide radicals into H_2_O_2_ and O_2_ and protect body from oxidative stress. Our results showed that Ni was low in patients suffering from RA while some found Ni level was in normal range (20) and some other authors reported high level of Ni in blood of RA patients as compared to control (21). Gender base comparison showed significant difference for Cr and Ni level in serum of RA patients (*P*<0.05) while non-significant difference exist for Cr and Ni level in RA patients (*P*>0.01) (21).

Correlation studies revealed highly significant but positive correlation between Cd-Cr, Cd-Pb and negative between Cr-Pb in RA patients while negative correlation was reported between Cr-Pb, Cd-Pb and positive between Cd-Cr in RA patients (*P*<0.01) (18). In contrast, healthy control individuals showed negative correlation between Cd-Cr, Cd-Pb and positive between Cr-Pb while opposite findings were reported for control individuals (18).

Our results were different from the published ones due to some natural factors viz. genetic Race, climate changes, demographic factors etc.

## Conclusion

Concentration of heavy metals in serum samples of RA patients and healthy control individuals differ significantly which shows that heavy metals may have contribution towards development of RA.

## Ethical considerations

Ethical issues (Including plagiarism, informed consent, misconduct, data fabrication and/or falsification, double publication and/or submission, redundancy, etc.) have been completely observed by the authors.
